# Variations in prevalence of retinopathy of prematurity (ROP) and completeness of screening in five units within a South African region: a register-based study

**DOI:** 10.1038/s41433-026-04257-y

**Published:** 2026-02-17

**Authors:** Tshilidzi Van der Lecq, Gerd Holmström, Esmè Jordaan, Phumza Nongena, Natasha Rhoda, Rudzani Muloiwa

**Affiliations:** 1 Department of Surgery, Division of Ophthalmology, Groote Schuur Hospital, University of Cape Town, Cape Town, South Africa; 2 Department of Surgical Sciences, Ophthalmology, Uppsala University, Uppsala, Sweden; 3 Biostatistics Research unit, South African Medical Research Council, Cape Town, South Africa; 4 Statistics and Population Studies Department, University of the Western Cape, Cape Town, South Africa; 5 Department of Paediatrics & Child Health, New Somerset Hospital, University of Cape Town, Cape Town, South Africa; 6 Department of Paediatrics & Child Health, Mowbray Maternity Hospital, University of Cape Town, Cape Town, South Africa; 7 Department of Paediatrics & Child Health, Red Cross War Memorial Children’s Hospital, University of Cape Town, Cape Town, South Africa; 8 Division of Ophthalmology, Stellenbosch University and Tygerberg Hospital, Cape Town, South Africa; 9 Department of Surgery, Division of Ophthalmology, Red Cross War Memorial Children’s Hospital, University of Cape Town, Cape Town, South Africa; 10 Department of Paediatrics and Child Health, Groote Schuur Hospital, University of Cape Town, Cape Town, South Africa; 11 Department of Paediatrics and Child Health, Paarl Hospital, Stellenbosch University, Cape Town, South Africa; 12 Division of Neonatology, Department of Paediatrics and Child Health, Stellenbosch University, Cape Town, South Africa

**Keywords:** Retinal diseases, Paediatrics, Epidemiology

## Abstract

**Background:**

Retinopathy of prematurity (ROP) is an avoidable cause of childhood blindness. Effective ROP screening enables the detection of cases requiring treatment, but the frequency of ROP varies in units across South Africa (SA). Recent evidence suggests that a high proportion of infants in SA do not complete screening. A comparison of ROP prevalence and completeness of screening in units within the same region has yet to be described.

**Methods:**

A prospective study of infants screened for ROP at five units was conducted between 1 February 2023 and 30 April 2024 in Cape Town, SA. Data were extracted for infants with birth weight <1250 grams or gestational age <32 weeks from the Retinopathy of Prematurity South African (ROPSA) register.

**Results:**

Screening was initiated in 933 infants and completed in 468 (50.2%). Completeness of screening varied in the units from 36.8% to 74.6%. The prevalence of ROP among infants who started screening (16.1%–71.2%), completed screening (13.1%–64.3%), and those incompletely screened (25.0%–79.2%) also varied in the units. Among those diagnosed with ROP (*n* = 315), 179 (56.8%) had incomplete screening, including 30 (*n* = 51, 58.8%) infants with stage 3 ROP.

**Conclusion:**

Completeness of screening and ROP prevalence varied widely in the units. Of concern is the high proportion of infants with ROP, including stage 3 ROP, who do not complete screening. Measures to increase completeness of screening are needed to improve the detection of cases requiring treatment. The ROPSA register will allow these improvements to be monitored over time.

## Introduction

Retinopathy of prematurity (ROP) is one of the most common avoidable causes of childhood blindness [1]. Low- and middle-income countries (LMICs), including those in Sub-Saharan Africa (SSA) are still experiencing what is termed the third epidemic of childhood blindness due to ROP [2]. Establishing efficient ROP screening programs to allow timely diagnosis and treatment of at risk infants is a key strategy to reduce childhood blindness due to ROP [3].

A 2018 global survey assessing the availability of ROP screening indicated little evidence for the presence of established ROP screening programs in most African countries [4]. Subsequent to this study, some SSA countries (i.e. Botswana, Ethiopia, Uganda, Tanzania) have established ROP screening programs and published data on the burden/frequency of ROP [5–8]. South Africa (SA) has regularly published data from several units over the past few decades. These studies report a wide range of ROP frequencies from 12.1% to 33.4% among screened preterm infants [9–18]. However, most of these studies do not comment on completeness of screening or the proportion of infants diagnosed with ROP who do not complete screening.

Incomplete ROP screening has been identified as a challenge in high, middle and low-income settings [19–21]. In the recently established South African ROPSA register, up to 46% of infants who initiated screening in the Cape Town Metropole region did not complete screening [22]. The study raised concerns that this may underestimate the true prevalence of ROP and treatment requiring ROP. Most studies report the frequency of ROP among infants who complete screening and exclude data from infants in whom screening is initiated but not completed. Using data from the ROPSA register, this study aims to compare the characteristics of infants who start screening and describe the prevalence of ROP in infants with complete and incomplete screening at five units within the Cape Town Metropole region.

## Subjects and methods

### Study population

This prospective population-based study included infants screened for ROP at three tertiary-level units (A, B and C) and two secondary-level units (D and E) within the Cape Town Metropole region of the Western Cape, South Africa [23]. These units are responsible for ROP screening in the public sector of the Cape Town Metropole, which serves 75% of a regional population of 4.7 million [23, 24]. Importantly, these units share similar features: they have adopted similar supplemental oxygen therapy protocols for preterm infants, including target oxygen saturation levels set at 90–94/95%; they all have access to key neonatal care resources, including continuous positive airway pressure, oxygen blenders, and surfactant; and they use the same ROP screening criteria, guidelines and case definition of treatment requiring ROP (Type 1 ROP).

## Data management

Data on all infants born and screened at the five units between 1 February 2023 and 30 April 2024 were extracted from the ROPSA register and analysed [22]. In most cases, screening was initiated where infants were born during admission [22]. Some infants were however, transferred between units for neonatal care purposes (i.e. Unit C to B or D vs Unit A to E). To prevent duplicate entries, infants were allocated to the unit that initiated screening even if follow-up examinations were subsequently performed at another unit. Demographic, neonatal, and ROP screening data were extracted for all preterm infants screened according to the regional screening criteria of birth weight (BW) < 1250 grams or gestational age (GA) < 32 weeks.

## Screening procedure

ROP screening was initiated at a postnatal age (PNA) of 4–6 weeks or postmenstrual age (PMA) of 31–33 weeks, whichever came latest [25]. This is in keeping with the National South African guidelines, which were amended from those used in the United Kingdom and the United States. Topical 2.5% cyclopentolate and 0.5% phenylephrine were instilled to dilate the pupils prior to screening. Screening at all the units was performed using binocular indirect ophthalmoscopy and a 28-dioptre lens. Examinations were performed by ophthalmologists or supervised ophthalmology trainees weekly or twice weekly. Based on the SA guidelines the screening process was noted as complete in infants with (a) full retinal vascularisation, or (b) completely regressed ROP, or (c) vascularisation to zone 3 without previous zone 1 or 2 ROP, or (d) attaining 45 weeks PMA with no Type 1 or Type 2 ROP disease or worse [25]. In addition, all infants diagnosed with treatment requiring ROP were considered to have completed screening.

In all five units, similar standardised templates were used to record retinal findings. These findings were described according to the International Classification of ROP guidelines (ICROP 3) [26]. The most advanced ROP stage diagnosed, in either eye, was assigned to each infant for analysis. Treatment-requiring ROP (or Type 1 ROP) was defined as (a) any stage of ROP in zone 1 with plus disease, or (b) stage 3 in zone 1 without plus disease, or (c) stage 2 or 3 ROP with plus disease in zone 2 [27].

## Statistical analysis

The analyses were performed using SAS v9.4 statistical software (SAS Institute Inc., Cary, NC: SAS Institute Inc.). Infants screened at or prior to 6 weeks PNA or 33 weeks PMA were considered to be screened on time.

The categorical covariates (sex, gestation, the number screened on time, the number who completed screening, no ROP, any ROP and ROP stages) were described using the number (*n*,%) of infants. Proportional differences between units were analysed using Chi-square tests.

Birth weight (grams) and gestational age (weeks) data were described using means with standard deviations and ranges. Birth weight was log-transformed to normalise the distribution. The unit means were compared using ANOVA and *t*-tests for BW (log) and GA with overall *p* values for Chi-Square tests. Pairwise comparisons between unit *p* values were reported for *z* tests adjusted for the multiple comparisons (Tukey-Kramer).

The frequency of ROP was compared in infants with complete and incomplete screening using a generalised linear model with a binomial distribution and a log link. A *p* value of <0.05 indicated statistical significance. The *p* value for the overall difference was reported using a Chi-Square test and pairwise differences within units were reported using a *z* test.

## Ethics

Ethical approval for this study and the ROPSA register was provided by the University of Cape Town's Health research ethics committee (HREC 346/2021 and R017/2021) and conducted based on the Declaration of Helsinki. A waiver of consent was obtained for the analysis of the anonymised dataset.

## Results

Screening for ROP in the region was initiated in 933 infants during the study period. Among these infants, there were 484 (51.9%) females and 752 (81.1%) singletons. In this total study group, the mean GA was 28.8 (SD 1.7) weeks with a mean BW of 1102.2 (SD 240.7) grams. The GA and BW of infants screened in the five different units are presented in Table 1. The tertiary units (A, B and C) screened 809 (86.7%) infants.

**Table 1:** Characteristics of infants in the total study group (*n* = 933), by unit.

		All infants	Unit A	Unit B	Unit C	Unit D	Unit E	*P* value
Total, *n* (%)		933 (100)	473 (50.7)	205 (22.0)	131 (14.0)	72 (7.7)	52 (5.6)	-
Sex, *n* (%)	Female	484 (51.9)	245 (51.8)	100 (48.8)	68 (51.9)	43 (59.7)	28 (53.9)	0.616
	Male	449 (48.1)	228 (48.2)	105 (51.2)	63 (48.1)	29 (40.3)	24 (46.2)	
Gestation, *n* (%)	Singleton	752 (81.1)	382 (81.4)	164 (80.4)	98 (74.8)	61 (85.9)	47 (90.4)	0.098
	Multiple	175 (18.9)	87 (18.6)	40 (19.6)	33 (25.2)	10 (14.1)	5 (9.6)	
Birth weight (grams)	Mean birth weight, SD	1102.2, 240.7	1041.5^a^, 184.8	1243.5^a^, 292.2	1067.6^a^, 224.1	1171.7, 291.2	1087.7^a^, 174.4	<0.001
Gestational age (weeks)	Mean gestational age, SD	28.8, 1.7	28.6^b^, 1.6	29.4^b^, 1.9	28.6^b^,1.7	28.9, 1.8	28.9, 1.6	<0.001
Screening on time, *n* (%)	PNA ≤ 6 weeks or PMA ≤ 33 weeks	749 (80.3)	383 (81.0)	164 (80.0)	110 (84.0)	51 (70.8)	41 (78.9)	0.279

*SD* standard deviation, *PNA* postnatal age, *PMA* postmenstrual age.

^a^Pairwise significant difference between unit B versus 3 units (A, C, E); (*p* < 0.001).

^b^Pairwise significant difference between unit B versus 2 units (A, C); (*p* < 0.001).

**Fig. 1:**
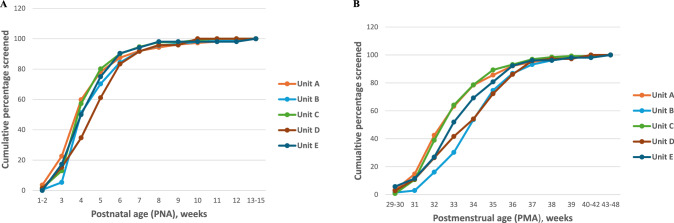
Timing of first screening examination at the units. Postnatal age (PNA)at first screening by unit (**A**) and Postmenstrual age at first screening by unit (**B**).

Screening in the region was initiated on time according to the guidelines, in 749 (80.3%) infants. The proportion of infants screened on time (i.e. at or prior to a PNA of 6 weeks or PMA of 33 weeks) in the five units ranged from 70.8% to 83.9% (*p* = 0.279). Screening in the units started between a PNA of 1–15 weeks and a PMA of 29–48 weeks. The cumulative percentage of infants screened at each PNA and PMA are illustrated in Fig. 1A, B.

## Completed screening

A total of 468 (50.2%) infants completed screening in the region. The proportion of infants in each unit who completed screening ranged from 36.8% to 74.6% (Table 2).

**Table 2:** Characteristics of infants who completed screening, by unit (*n* = 468, *N* = 933).

		All infants	Unit A	Unit B	Unit C	Unit D	Unit E	*P* value
Completeness of screening, n/N, %		468/933 50.2%	174/473, 36.8%^a^	153/205, 74.6%^a^	76/131, 58.0%^a^	37/72, 51.4%^a^	28/52, 53.8%^a^	<0.001
Birth weight (grams)	Mean Birth Weight, SD	1142.7, 258.4	1067.0^b^, 171.8	1254.8^b^, 298.4	1091.5^b^, 245.5	1270.5^b^, 288.7	1095.0, 153.7	<0.001
Gestational age (weeks)	Mean Gestational age, SD	29.0, 1.8	28.6^c^, 1.7	29.4^c^, 1.9	28.7^c,^ 1.6	29.7^c^, 1.5	28.9, 1.6	<0.001

*SD* standard deviation.

^a^Units C, D, E had similar percentages and are significantly different from the other units (*p* < 0.015).

^b^Units B&D were different from units A and C; (*p* < 0.004).

^c^Units B&D were different from units A and C; (*p* < 0.007).

The mean GA and BW in those that completed screening in the region was 29.0 (SD 1.8) weeks and 1142.7 (SD 258.4) grams, respectively. The mean GA of infants in the various units who completed screening ranged from 28.6 to 29.7 weeks, with the mean BW ranging from 1067.0 to 1270.5 grams; (*p* < 0.001, *p* < 0.001) (Table 2).

## Diagnosis of ROP among the screened cohorts

Among the 933 infants in the region in whom screening was initiated, 315 (33.8%, 95% CI 30.9%–36.9%) were diagnosed with ROP. The proportions of infants diagnosed with ROP in Unit A-E were 33.4% (158/473), 16.1% (33/205), 39.7% (52/131), 48.6% (35/72), and 71.2% (37/52), respectively (*p* < 0.001 for overall difference) (Table 3). Further, a maximum of stage 1 or 2 was diagnosed in 264 (28.3%) and stage 3 in 51 (5.5%) of the infants. Type 1 ROP was diagnosed in 16 (1.7%) infants. One infant demised prior to treatment and the other 15 were treated. No infants were diagnosed with stage 4 or 5 ROP.

**Table 3:** Prevalence of retinopathy of prematurity in all infants screened (*n* = 933), infants with complete (*n* = 468), and infants with incomplete screening (*n* = 465), by unit.

		Overall *N* = 933	Completed screening *N* = 468	Incomplete screening *N* = 465	*P* Value^*^
Unit A	Total *n* (%)	473 (100%)	174 (36.8)	299 (63.2)	0.703
	ROP *n*, % (95% CI)	158, 33.4 (29.4–37.9)	60, **34.5 (28.0–42.0)**	98, **32.8 (27.9–38.6)**	
Unit B^a^	Total *n* (%)	205 (100%)	153 (74.6)	52 (25.4)	0.042
	ROP *n*, % (95% CI)	33, 16.1 (11.8–22.0)	20, **13.1 (8.7–19.7)**	13, **25.0 (15.6–40.0)**	
Unit C	Total *n* (%)	131 (100%)	76 (58.0)	55 (42.0)	0.130
	ROP *n*, % (95% CI)	52, 39.7 (32.1–49.0)	26, **34.2 (25.1–46.7)**	26, **47.3 (35.8–62.5)**	
Unit D	Total *n* (%)	72 (100%)	37 (51.4)	35 (48.6)	0.008
	ROP *n*, % (95% CI)	35, 48.6 (38.3–61.6)	12, **32.4 (20.4–51.6)**	23, **65.7 (51.7–83.5)**	
Unit E^b^	Total *n* (%)	52 (100%)	28 (53.8)	24 (46.1)	0.236
	ROP *n*, % (95% CI)	37, 71.2 (59.9–84.6)	18, **64.3 (48.8–84.7)**	19, **79.2 (64.5–97.2)**	

*p value for comparison of ROP in completed screening and incomplete screening groups within each unit.

^a^unit B was lower than all other units; (*p* < 0.001).

^b^unit E was higher than unit A and C; (*p* < 0.001).

In the 468 (50.2%) infants who completed screening, 136 (29.1%) were diagnosed with ROP. A maximum of stage 1 or 2 was diagnosed in 115 (24.6%) and stage 3 in 21 (4.6%) of these infants. The proportion of these infants diagnosed with ROP in the units ranged from 13.1% to 64.3% (Table 3).

Overall, 465 (49.8%) infants in the region had incomplete screening. The proportion of infants with incomplete screening in each unit ranged from 25.4% to 63.2% (Table 3). Among these infants, 179 (38.5%) were diagnosed with ROP. A maximum of stage 1 or 2 was diagnosed in 149 (32.0%) and stage 3 in (30, 6.5%). The proportion of these infants diagnosed with ROP in the units ranged from 25.0% to 79.2% (Table 3).

Among the infants diagnosed with ROP in this region (*n* = 315), 179 (56.8%) had incomplete screening. This included 56.4% (149/264) of those diagnosed with a maximum of stage 1 and 2 and 58.8% (30/51, 58.8%) of those with a maximum of stage 3.

The reason for incomplete screening in the region was failure to attend scheduled follow-up examinations in 446 (95.9%), death in 15 (3.2%), referral outside of the region in 2 (0.4%), with no reason provided for 2 (0.4%) infants. Failure to attend follow-up examinations was also the most common reason for incomplete screening in Unit A-E: 95.6% (289/299), 100% (52/52), 92.7% (51/55), 94.3% (33/35), 100% (24/24), respectively.

## Discussion

To our knowledge, this is the first population-based study to describe the prevalence of ROP and completeness of screening in units within the same region. The five units within this Sub-Saharan African region demonstrated a wide variation in the prevalence of ROP and completeness of screening.

Screening was initiated on time in 80% of infants in this region, an encouraging finding when compared to recent population-based studies in India (43%, *n* = 751), the Netherlands (78.2%, *n* = 849), and France (75.7%, *n* = 2 169) [28–30]. Timely initiation of screening enables the diagnosis of ROP prior to progression to advanced stages.

The prevalence of ROP can vary within a country [7, 31, 32]. Our study found that ROP prevalence varies in units within the same region. While these units have adopted similar ROP screening and neonatal protocols, differences in adherence to these protocols may account for the different ROP prevalences. Another explanation is the difference in the characteristics (i.e. GA and BW) of infants screened in the units. Lower GAs and BWs are associated with an increased risk of developing ROP [33]. Due to regional neonatal policy, less mature preterm infants are born in tertiary Units A and C. The finding that Unit E, a secondary unit, had the highest proportion of infants diagnosed with ROP was surprising. However, the GA and BW of the screened infants were similar to tertiary Unit A and C. In the ROPSA register infants are assigned to the unit which starts ROP screening. Some infants may have been transferred from tertiary Unit A, with ROP screening initiated in Unit E. The diagnosis of ROP in 64% of infants completing screening in Unit E is the highest reported by any unit in South Africa [9–18]. Infants transferred from tertiary to secondary level care prior to completed screening may have an increased risk of developing ROP in this region. Currently, the ROPSA register does not collect data on the transfer of patients between units. Data on transfers may be useful in this region, especially since transfer between units before starting or completing ROP screening increases the risk of incomplete screening [30, 34, 35].

Incomplete screening is reported to be as high as 46% (*n* = 318/696) in the Cape Town Metropole region [22]. The present study confirmed this high level of incomplete screening in the region (50%, *n* = 465/933) and identifies a wide variation in the different units within this region, as low as 25% (Unit B, *n* = 52/205) and as high as 63% (Unit A, *n* = 299/473). These figures are higher than other SSA units: 8.8% (*n* = 12/135) [16], 10% (*n* = 53/526) [11], 22% (27/121) [12] in SA versus 13% (*n* = 54/424) [36], 21% (*n* = 52/245) [8] in other African countries. This is the first SA study to explore reasons for incomplete screening. Failure to attend scheduled follow-up examinations was the most common reason in the units and the region. A recent study (*n* = 66), assessed different reasons for the failure to attend scheduled outpatient ROP screening examinations. The authors analysed the role of maternal ethnicity, median family income, GA and BW. The study found low birth weight to be the only significant factor associated with loss to follow-up [20]. Screening in the units was initiated on time for the majority of infants, most likely because infants were still inpatients. While data on the inpatient or outpatient status of infants at the time of screening is currently not collected on the ROPSA register, we suspect that outpatient appointments may contribute to incomplete screening in this region and underlying reasons need to be explored.

It is concerning that most (57%, 179/315) infants diagnosed with ROP in this region do not complete screening. This includes 59% (30/51) of those known to have developed stage 3 ROP. Based on the large randomised Early Treatment for Retinopathy of Prematurity study in the United States, infants with stage 3 ROP have a 12% -16% risk of progression to Type 1 ROP [37]. Improving completeness of screening will provide a more reliable estimate of the prevalence of ROP and Type 1 ROP in this region, which may be higher than previously estimated. More importantly, it will reduce the risk of missing infants that need treatment to reduce the risk of blindness [27]. Strategies to encourage adherence to follow-up examinations, especially in infants known to have developed ROP are needed. Measures that have worked in other LMICS include: counselling caregivers about the importance of screening, travel reimbursement for infants with severe ROP, recording alternative contact numbers for caregivers, and the appointment of a project manager to liaise with caregivers [38].

The strength of this study is the use of the ROPSA register which collects population level ROP screening data while monitoring the underlying screening process. This has allowed us to quantify infants with incomplete screening and the proportion of these infants diagnosed with ROP. This study highlights that the three tertiary units (A-C) screened almost 90% of the infants and diagnosed 15 of the 16 infants who developed Type 1 ROP. While Unit A initiates screening in half the infants in this region, an alarming 63% of these infants did not complete screening. Such data can be used to guide the allocation of resources to improve ROP screening at a regional level.

While this study analysed some risk factors for developing ROP (i.e. BW, GA, sex), the inclusion of additional risk factors was beyond the scope of this study. This is a limitation, since differences in risk factors may contribute the ROP prevalences in the units. Based on the ROPSA data these can be analysed in the future.

In conclusion, our findings confirm that a large proportion of infants in this South African region do not complete ROP screening, and this varies in the units. The current prevalence of ROP may be underestimated in these units which provide ROP screening to most infants in this region. Sustaining the ROPSA register with the addition of key data will allow continuous monitoring of completeness of screening, ROP frequency, and aid in identifying reasons for the observed differences. Given the wide variability in prevalence, it is important that data should be collected from every screening neonatal unit. Sampling from only some of the units, especially only tertiary, is likely to underestimate the true burden of ROP.

Our findings will be shared with stakeholders in the different units. Measures to improve completeness of screening will need to be identified and implemented to optimise the care and visual outcome in preterm infants in this region.

## Summary

### What was known before:

- The prevalence of retinopathy of prematurity can vary within a country.

### What this study adds:

- The prevalence of retinopathy of prematurity can vary in units located within the same region; regardless of similarities in neonatal care and screening protocols.

## Data Availability

The data from the study are available on reasonable request from the corresponding author.
